# Supplementary material for: Relationships between perceived measures of internal load and wellness status during overseas futsal training camps
Source: PLoS One. 2022 Apr 19;17(4):e0267227. doi: 10.1371/journal.pone.0267227 (PMC9017953; doi:10.1371/journal.pone.0267227)
Supplement: Attachment — Submitted filename: Authors responses to reviewers 2_PONE-D-21-37878.docx [file pone.0267227.s003.docx]

**Plos One**

Manuscript number: PONE-D-21-37878

Title: Relationship between Relationships between perceived internal training loads and psychometric wellness during overseas futsal training camps

Dear Reviewer:
Thank you very much for your suggestions and the valuable comments. We have revised the manuscript according to the reviewers’ comments. We provide point-by-point responses (in RED color) to the reviewers’ comments below. Revision in responses to each question/concern raised by the reviewers are noted with tracked changes in the manuscript.

REVIEWER 2

The study investigated the effects of different OTC on TL and well being measures as well as their relationships. The study is of interest and the authors did good job. There are some issues to be addressed, and a general English revision is required before the formal acceptance of the paper.

Response: Thank you very much for your supportive comment and valuable suggestions. We have improved the quality of the paper, according to the reviewers’ comments. Additionally, a native English speaker with good knowledge in sports sciences has provided feedback and proofread the revised manuscript.

Specific comments
Abstract
L49: the authors said that the relationship between TL and recovery status has not been established during short-term overseas training camps (OTC). However, previous study (Lu et al., 2018) has investigated such relationship during OTC? Any clarifications? Please remove it or just say that it was not extensively investigated to not neglect previous research.

Response: Thank you for the suggestion. We have now revised the sentence, according to your comments in lines 49-51.

- Please add effect size’s values for different comparisons and r values for correlations.

Response: Thank you for your valuable comment. We added ES and r value of correlation output in the abstract as per your comment.

Introduction
This part is well written by the authors.

Response: Thank for your positive comment.

Why the authors did not report that one previous study also investigated the relationship between TL and recovery state in OTC (https://www.termedia.pl/Training-load-and-recovery-status-during-a-short-term-overseas-training-camp-in-Taiwan-futsal-players,132,35932,0,0.html). I think it is important for your paper to clear for readers what is different from this one and this strengthens your rationale.

Response: Thank for your valuable comment. We added a sentence to address your concern. Line 125-127.

Materials and Methods
Participants
L138: “called up” may be appropriate to change to “recruited “

Response: Revised accordingly. Line 159.

- A sample size calculation is required here, please add it.

Response: Thank for your valuable comment. We agree with the reviewer that sample size estimation is critical to examine the power of the results. However, this study is an observational study, based on the training schedule and team preparation of a national team. This study was therefore based on a sample of convenience and thus the number of players varied from camp to camp. We will keep this factor in mind for preparing overseas training camps in the future.

- Please provide psychometric properties for measures used

Response: We apologize for the misleading statement for psychometric properties. To avoid the potential for confusion, we have remove the term “psychometric” and in its place inserted wellness status in the revision. Line 195-203.

Discussion
The discussion is written well.

Response: Thank you for your positive comment.

Figure
I would ask the authors to change the figure as it is not clear.

Response: Thank you for your comment. We apologized for the low-resolution of figure 1 in the manuscript. After double checking, the figure 1 upload to the submission matches the resolution requirement of Plos One (300 dpi). However, our originally submission was a JPG file. We have converted to a TIFF file as the journal requires.
