# Supplementary material for: Relationships between perceived measures of internal load and wellness status during overseas futsal training camps
Source: PLoS One. 2022 Apr 19;17(4):e0267227. doi: 10.1371/journal.pone.0267227 (PMC9017953; doi:10.1371/journal.pone.0267227)
Supplement: Attachment — Submitted filename: Authors responses to reviewers 2_PONE-D-21-37878_R2.docx [file pone.0267227.s004.docx]

**Plos One**

Manuscript number: PONE-D-21-37878

Title: Relationships between perceived measures of internal load and wellness status during overseas futsal training camps

Dear Reviewer:
Thank you very much for your suggestions and the valuable comments.

REVIEWER 2

I congratulate the authors as they addressed almost of my comments satisfactorly. I recommend the paper to be accepted.

Responses: Thank you very much for your time and effort to improve the quality of the manuscript. Thanks again for your support.
